# Integrated genomic view of SARS-CoV-2 in India

**DOI:** 10.12688/wellcomeopenres.16119.1

**Published:** 2020-08-03

**Authors:** Pramod Kumar, Rajesh Pandey, Pooja Sharma, Mahesh S. Dhar, Vivekanand A., Bharathram Uppili, Himanshu Vashisht, Saruchi Wadhwa, Nishu Tyagi, Saman Fatihi, Uma Sharma, Priyanka Singh, Hemlata Lall, Meena Datta, Poonam Gupta, Nidhi Saini, Aarti Tewari, Bibhash Nandi, Dhirendra Kumar, Satyabrata Bag, Deepanshi Gahlot, Surabhi Rathore, Nidhi Jatana, Varun Jaiswal, Hema Gogia, Preeti Madan, Simrita Singh, Prateek Singh, Debasis Dash, Manju Bala, Sandhya Kabra, Sujeet Singh, Mitali Mukerji, Lipi Thukral, Mohammed Faruq, Anurag Agrawal, Partha Rakshit

**Affiliations:** 1 Biotechnology Division, National Centre for Disease Control, Delhi, Delhi, 110054, India; 2 Department of Genomics and Molecular Medicine, CSIR-Institute of Genomics and Integrative Biology, Delhi, Delhi, 110007, India

**Keywords:** SARS-CoV-2, COVID-19, COVID, viral genomes, MinION, Whole Genome Sequencing

## Abstract

**Background: **India first detected SARS-CoV-2, causal agent of COVID-19 in late January 2020, imported from Wuhan, China. From March 2020 onwards, the importation of cases from countries in the rest of the world followed by seeding of local transmission triggered further outbreaks in India.

**Methods: **We used ARTIC protocol-based tiling amplicon sequencing of SARS-CoV-2 (n=104) from different states of India using a combination of MinION and MinIT sequencing from Oxford Nanopore Technology to understand how introduction and local transmission occurred.

**Results: **The analyses revealed multiple introductions of SARS-CoV-2 genomes, including the A2a cluster from Europe and the USA, A3 cluster from Middle East and A4 cluster (haplotype redefined) from Southeast Asia (Indonesia, Thailand and Malaysia) and Central Asia (Kyrgyzstan). The local transmission and persistence of genomes A4, A2a and A3 was also observed in the studied locations. The most prevalent genomes with patterns of variance (confined in a cluster) remain unclassified, and are here proposed as A4-clade based on its divergence within the A cluster.

**Conclusions: **The viral haplotypes may link their persistence to geo-climatic conditions and host response. Multipronged strategies including molecular surveillance based on real-time viral genomic data is of paramount importance for a timely management of the pandemic.

## Introduction

The ongoing pandemic of COVID-19 caused by SARS-CoV-2 following its first appearance in China has pressed the global community to take measures to flatten its transmission ( Chan *et al.*, 2020; Zhu *et al.*, 2020). The severe symptoms of infection can include pneumonia, severe acute respiratory syndrome, kidney failure and even death with a coalescence of factors ( Young *et al.*, 2020; Zhu *et al.*, 2020). Many COVID-19 cases have been reported to be asymptomatic and may serve as carrier of SARS-CoV-2 ( He *et al.*, 2020; Xu *et al.*, 2020). Genome sequences of SARS-CoV-2 suggest its origin and transmission patterns after it enters a new population is proving to be an important step towards formulating strategies for management of this pandemic ( Andersen *et al.*, 2020; Chen & Li, 2020).

The first three cases in India were reported in late January and early February, in individuals with a travel history of Wuhan, China. India took drastic steps to contain the further spread of the virus including imposition of travel restrictions to and from the affected countries. There were no new cases of COVID-19 for almost a month. However, while the global focus was on China and other eastern countries like South Korea and Japan; European countries, the Middle East and the USA reported a surge in cases of COVID-19. March 2020 onwards, India also witnessed a surge of imported cases from countries other than China which has been further assisted with local transmission. In March, imposition of nationwide lockdown checked the epidemic curve. Despite these measurements, India is at the verge of a large outbreak as the transmission is rapidly increasing with more than 450,000 reported cases of COVID-19 by the fourth week of June 2020.

We carried out whole genome sequencing of SARS-CoV-2 (n=104) from Pan-India through Surveillance Program of the National Center for Disease Control (NCDC), Delhi. Here, we combine genetic and epidemiological data to understand the genetic diversity, evolution, and epidemiology of SARS-CoV-2 across India. The spectrum of variations would be an important tool towards contact tracing, effective diagnostics and backbone for drug and vaccine development.

## Methods

### Subject recruitment

The study was conducted jointly by the NCDC and CSIR-Institute of Genomics and Integrative Biology (CSIR-IGIB). Institutional ethical clearance was obtained at both institutes prior to initiation of research; the need for consent from the patients was waived by the committee. A total of 127 laboratory-confirmed cases of COVID-19 from targeted testing and available samples at NCDC which represent different geographic locations or states and travel history from different countries during the early phase of the outbreak ( Table 1 and *Extended data*, Supplementary figure S1 [ Kumar *et al.*, 2020b]). were included in the study for genomic analyses. Targeted testing involved suspected cases; having symptoms (fever, cough and breathlessness) with recent travel history to high-risk countries (China, South Asia, Middle East, European countries such as Italy, Spain, UK, France and USA) or positive contacts of COVID-19 cases.

**Table 1.** Frequency and description the variations obtained in the SARS-CoV-2 genome from major identified cluster from 104 sequences.

							Global Frequency					
**POS**	**Gene**	**Gene**	**clade**	**Aminoacid** **change**	**Amino Acid changes**	**effect**	**A**	**T**	**G**	**C**	**N**	**Variant** **count** **in this** **study**
**11083**	NSP6	orf1ab	A3	Leu3606Phe	QHD43415.1:p.3606L; QHD43415.1:p.3606L>F; QHD43415.1:p.3606-	LOW; MODERATE; MODIFIER	0	1137	7544	0	41	68
**13730**	NSP12/RdRP	orf1ab	-	Ala4489Val	QHD43415.1:p.4489A>V	MODERATE	0	99	0	8622	1	65
**6312**	NSP3	orf1ab	-	Thr2016Lys	QHD43415.1:p.2016T>K; QHD43415.1:p.2016T>I; QHD43415.1:p.2016T>R	MODERATE	93	3	0	8625	1	62
**28311**	N-capsid	N	-	Pro13Leu	QHD43423.2:p.13P>L; QHD43423.2:p.13P>R; QHD43423.2:p.13-	MODERATE; MODIFIER	0	160	1	8560	1	53
**23929**	S-Protein	S	-	Tyr789Tyr	QHD43416.1:p.789Y; QHD43416.1:p.789-	LOW; MODIFIER	0	88	0	8627	7	46
**14408**	NSP12/RdRP	orf1ab	A2a	Pro4715Leu	QHD43415.1:p.4715P>L; QHD43415.1:p.4715-	MODERATE; MODIFIER	0	5325	0	3381	16	26
**23403**	S-Protein	S	A2	Asp614Gly	QHD43416.1:p.614-; QHD43416.1:p.614D>G	MODIFIER; MODERATE	3346	0	5356	0	20	26
**241**	5'UTR	5'UTR	A2	0	QHD43415.1	MODIFIER,DISTANCE=25	0	5194	0	3149	379	24
**3037**	NSP3	orf1ab	A2	Phe924Phe	QHD43415.1:p.924F; QHD43415.1:p.924-	LOW; MODIFIER	1	5348	0	3352	21	23
**6310**	NSP3	orf1ab	-	Ser2015Arg	QHD43415.1:p.2015S>R; QHD43415.1:p.2015-; QHD43415.1:p.2015S	MODERATE; MODIFIER; LOW	28	17	0	8672	5	22
**25563**	ORF3a	ORF3a	A2a2	Gln57His	QHD43417.1:p.57Q>H; QHD43417.1:p.57-	MODERATE; MODIFIER	0	2300	6415	1	6	9
**1397**	NSP2	orf1ab	A3	Val378Ile	QHD43415.1:p.378V>I	MODERATE	137	0	8584	0	1	7
**18877**	NSP14/Exonuclease	orf1ab	-	Leu6205Leu	QHD43415.1:p.6205L	LOW	0	208	0	8512	2	7
**884**	NSP2	orf1ab	-	Arg207Cys	QHD43415.1:p.207R>C	MODERATE	0	39	0	8682	1	6
**8653**	NSP4	orf1ab	-	Met2796Ile	QHD43415.1:p.2796M>I	MODERATE	0	37	8680	0	5	6
**26735**	M-Protein	M	-	Tyr71Tyr	QHD43419.1:p.71Y; QHD43419.1:p.71-	LOW; MODIFIER	0	41	0	8677	4	6
**28688**	N-capsid	N	A3	Leu139Leu	QHD43423.2:p.139L; QHD43423.2:p.139-	LOW; MODIFIER	0	8586	0	130	6	6
**29742**	3'UTR	3'UTR	A3	0	QHI42199.1	MODIFIER,DISTANCE=68	61	120	8268	0	273	6
**12685**	NSP8	orf1ab	-	Gln4140His	QHD43415.1:p.4140Q>H; QHD43415.1:p.4140-	MODERATE; MODIFIER	0	0	8720	0	2	5
**16993**	NSP13/Helicase	orf1ab	-	Tyr5577His	QHD43415.1:p.5577Y>H	MODERATE	0	8715	0	6	1	5
**22444**	S-Protein	S	-	Asp294Asp	QHD43416.1:p.294D	LOW	0	13	0	8438	271	5
**25461**	ORF3a	ORF3a	-	Ala23Ala	QHD43417.1:p.23A	LOW	0	8714	0	6	2	5
**28854**	N-capsid	N	-	Ser194Leu	QHD43423.2:p.194S>L; QHD43423.2:p.194-	MODERATE; MODIFIER	0	64	0	8654	4	5
**1706**	NSP2	#N/A	-	Ser481fs	#N/A	#N/A	0	8721	0	0	1	4
**1707**	NSP2	orf1ab	-	Ser481Phe	QHD43415.1:p.481S>F; QHD43415.1:p.481-	MODERATE; MODIFIER	0	3	0	8716	3	4
**658**	NSP1	orf1ab	-	Ala131Ala	QHD43415.1:p.131A; QHD43415.1:p.131-	LOW; MODIFIER	0	8706	0	14	2	3
**1820**	NSP2	orf1ab	-	Gly519Ser	QHD43415.1:p.519G>S; QHD43415.1:p.519-	MODERATE; MODIFIER	13	0	8708	0	1	3
**7621**	ORF1a/b	orf1ab	-	Cys2452Cys	QHD43415.1:p.2452C	LOW	0	8720	0	0	2	3
**8782**	NSP4	orf1ab	-	Ser2839Ser	QHD43415.1:p.2839S; QHD43415.1:p.2839-	LOW; MODIFIER	0	1370	0	7344	8	3
**14805**	NSP12/RdRP	orf1ab	A1a1	Tyr4847Tyr	QHD43415.1:p.4847Y; QHD43415.1:p.4847-	LOW; MODIFIER	0	892	0	7813	17	3
**18486**	NSP14/Exonuclease	orf1ab	-	Leu6074Leu	QHD43415.1:p.6074L	LOW	0	3	0	8717	2	3
**19524**	NSP14/Exonuclease	orf1ab	-	Leu6420Leu	QHD43415.1:p.6420L; QHD43415.1:p.6420-	LOW; MODIFIER	0	33	0	8501	188	3
**21792**	S-Protein	S	-	Lys77Met	QHD43416.1:p.77K>M	MODERATE	8717	0	0	0	5	3
**27191**	M-Protein	M	-	Ter223Ter	QHD43419.1:p.223-; QHD43419.1:p.223*	MODIFIER; LOW	8717	0	2	0	3	3
**28881**	N-capsid	N	A2a1	Arg203Lys	QHD43423.2:p.203R>K; QHD43423.2:p.203R>M; QHD43423.2:p.203-	MODERATE; MODIFIER	1357	1	7347	0	17	3
**28882**	N-capsid	N	A2a1	Arg203Arg	QHD43423.2:p.203R; QHD43423.2:p.203R>S; QHD43423.2:p.203-	LOW; MODERATE; MODIFIER	1353	0	7353	0	16	3
**28883**	N-capsid	N	A2a1	Gly204Arg	QHD43423.2:p.204-; QHD43423.2:p.204G>R	MODIFIER; MODERATE	0	0	7353	1353	16	3
**1059**	NSP2	orf1ab	A2a2a	-	QHD43415.1:p.265T>I; QHD43415.1:p.265-	MODERATE; MODIFIER	0	1900	0	6816	6	2
**1281**	NSP2	orf1ab	-	Ala339Val	QHD43415.1:p.339A>V; QHD43415.1:p.339-	MODERATE; MODIFIER	0	3	0	8717	2	2
**1947**	NSP2	#N/A	-	-	#N/A	#N/A	0	8720	0	0	2	2
**2480**	NSP2	orf1ab	-	Ile739Val	QHD43415.1:p.739-; QHD43415.1:p.739I>V	MODIFIER; MODERATE	8419	0	292	0	11	2
**2558**	NSP2	orf1ab	A1a1b	Pro765Ser	QHD43415.1:p.765P>S; QHD43415.1:p.765-	MODERATE; MODIFIER	0	321	0	8390	11	2
**7600**	ORF1a/b	orf1ab	-	Cys2445Cys	QHD43415.1:p.2445C; QHD43415.1:p.2445-	LOW; MODIFIER	0	0	0	8721	1	2
**13920**	NSP12/RdRP	#N/A	-	Lys4552Lys	#N/A	#N/A	0	0	8721	0	1	2
**16355**	NSP13/Helicase	orf1ab	-	Lys5364Arg	QHD43415.1:p.5364K>R	MODERATE	8721	0	0	0	1	2
**18395**	NSP14/Exonuclease	orf1ab	-	-	QHD43415.1:p.6044A>V	MODERATE	0	2	0	8719	1	2
**18803**	NSP14/Exonuclease	orf1ab	-	Ser6180Ile	QHD43415.1:p.6180S>I	MODERATE	0	0	8721	0	1	2
**19684**	EndoRNAse	orf1ab	-	Val6474Leu	QHD43415.1:p.6474V>L	MODERATE	0	43	8663	0	16	2
**21137**	O-ribose methyltransferase	orf1ab	-	Lys6958Arg	QHD43415.1:p.6958-; QHD43415.1:p.6958K>R	MODIFIER; MODERATE	8705	0	14	0	3	2
**22289**	S-Protein	S	-	Ala243Ser	QHD43416.1:p.243A>S	MODERATE	0	2	8716	0	4	2
**26144**	ORF3a	ORF3a	A1a	Gly251Val	QHD43417.1:p.251G>V; QHD43417.1:p.251-	MODERATE; MODIFIER	0	820	7890	0	12	2
**28144**	ORF8	ORF8	B	Leu84Ser	QHD43422.1:p.84L>S; QHD43422.1:p.84-	MODERATE; MODIFIER	0	7346	0	1353	23	2
**28144**	ORF8	ORF8	B	Leu84Ser	QHD43422.1:p.84L>S; QHD43422.1:p.84-	MODERATE; MODIFIER	0	7346	0	1353	23	2
**29555**	orf	Intergenic	-	0	QHI42199.1	MODIFIER,DISTANCE=3	0	1	0	8710	11	2
**29555**	orf	Intergenic	-	0	QHI42199.1	MODIFIER,DISTANCE=3	0	1	0	8710	11	2
**29729**	3'UTR	3'UTR	-	-	QHI42199.1	MODIFIER,DISTANCE=55	0	8457	1	0	264	2
**84**	5'UTR	5'UTR	-	-	0	MODIFIER	1	3	0	8137	581	1
**167**	5'UTR	5'UTR	-	-	QHD43415.1	MODIFIER,DISTANCE=99	0	1	8337	1	383	1
**203**	5'UTR	5'UTR	-	-	QHD43415.1	MODIFIER,DISTANCE=63	0	3	1	8355	363	1
**337**	NSP1	orf1ab	-	-	QHD43415.1:p.24R	LOW	0	2	0	8686	34	1
**507**	NSP1	orf1ab	-	-	QHD43415.1:p.81-86HGHVMV>H	MODERATE	8711	0	0	0	11	1
**509**	NSP1	orf1ab	-	-	QHD43415.1:p.82-85GHVM>V	MODERATE	0	0	8710	0	12	1
**561**	NSP1	#N/A	-	-	#N/A	#N/A	0	0	8716	0	6	1
**683**	NSP1	orf1ab	-	-	QHD43415.1:p.140-143LKSF>L; QHD43415.1:p.140L	MODERATE; LOW	0	3	0	8716	3	1
**851**	NSP2	orf1ab	-	-	QHD43415.1:p.196Y>H	MODERATE	0	8721	0	0	1	1
**1281**	NSP2	orf1ab	-	Ala339Val	QHD43415.1:p.339A>V; QHD43415.1:p.339-	MODERATE; MODIFIER	0	3	0	8717	2	1
**1601**	NSP2	orf1ab	-	-	QHD43415.1:p.446L>I	MODERATE	1	0	0	8719	2	1
**1601**	NSP2	orf1ab	-	-	QHD43415.1:p.446L>I	MODERATE	1	0	0	8719	2	1
**1887**	NSP2	orf1ab	-	-	QHD43415.1:p.541A>V; QHD43415.1:p.541-	MODERATE; MODIFIER	0	2	0	8717	3	1
**1912**	NSP2	orf1ab	-	-	QHD43415.1:p.549S	LOW	0	17	0	8703	2	1
**1912**	NSP2	orf1ab	-	-	QHD43415.1:p.549S	LOW	0	17	0	8703	2	1
**2040**	NSP2	orf1ab	-	-	QHD43415.1:p.592T>I	MODERATE	0	1	0	8719	2	1
**2485**	NSP2	orf1ab	-	-	QHD43415.1:p.740I	LOW	0	2	0	8719	1	1
**2558**	NSP2	orf1ab	A1a1b	Pro765Ser	QHD43415.1:p.765P>S; QHD43415.1:p.765-	MODERATE; MODIFIER	0	321	0	8390	11	1
**3145**	NSP3	orf1ab	-	-	QHD43415.1:p.960L>F	MODERATE	0	9	8710	0	3	1
**3176**	NSP3	orf1ab	-	-	QHD43415.1:p.971P>S	MODERATE	0	4	0	8712	6	1
**3429**	NSP3	orf1ab	-	-	QHD43415.1:p.1055T>I	MODERATE	0	1	0	8720	1	1
**3604**	NSP3	orf1ab	-	-	QHD43415.1:p.1113H	LOW	0	0	0	8721	1	1
**4144**	NSP3	#N/A	-	-	#N/A	#N/A	0	0	8718	0	4	1
**4680**	NSP3	#N/A	-	-	#N/A	#N/A	0	0	0	8721	1	1
**4859**	NSP3	#N/A	-	-	#N/A	#N/A	0	0	8720	0	2	1
**5008**	NSP3	orf1ab	-	-	QHD43415.1:p.1581T	LOW	0	0	8720	0	2	1
**5151**	NSP3	orf1ab	-	-	QHD43415.1:p.1629V>A	MODERATE	0	8720	0	0	2	1
**5657**	NSP3	orf1ab	-	-	QHD43415.1:p.1798V>I; QHD43415.1:p.1798V>L	MODERATE	1	0	8720	0	1	1
**5730**	NSP3	orf1ab	-	-	QHD43415.1:p.1822T>I	MODERATE	0	10	0	8711	1	1
**6395**	NSP3	orf1ab	-	-	QHD43415.1:p.2044L	LOW	0	3	0	8709	10	1
**7071**	NSP3	#N/A	-	-	#N/A	#N/A	1	0	8718	0	3	1
**7350**	ORF1a/b	#N/A	-	-	#N/A	#N/A	0	0	0	8720	2	1
**7734**	ORF1a/b	#N/A	-	-	#N/A	#N/A	0	8717	0	0	5	1
**8595**	NSP4	orf1ab	-	-	QHD43415.1:p.2777T>I	MODERATE	0	0	0	8721	1	1
**9477**	NSP4	orf1ab	-	-	QHD43415.1:p.3071F>Y	MODERATE	150	8567	0	0	5	1
**9634**	NSP4	orf1ab	-	-	QHD43415.1:p.3123L>F	MODERATE	8710	4	0	0	8	1
**10039**	NSP4	orf1ab	-	-	QHD43415.1:p.3258T; QHD43415.1:p.3258-	LOW; MODIFIER	0	1	0	8717	4	1
**10449**	Mpro	orf1ab	-	-	QHD43415.1:p.3395P>L; QHD43415.1:p.3395-	MODERATE; MODIFIER	0	3	0	8717	2	1
**11074**	NSP6	orf1ab	-	-	QHD43415.1:p.3604F>X; QHD43415.1:p.3603F; QHD43415.1:p.3603F>FX;	HIGH; LOW; MODERATE; MODIFIER	0	22	0	86	3	1
**11109**	NSP6	orf1ab	-	-	QHD43415.1:p.3615A>V	MODERATE	0	20	0	8699	3	1
**12929**	NSP9	orf1ab	-	-	QHD43415.1:p.4222-	MODIFIER	0	0	8720	0	2	1
**13038**	NSP10	#N/A	-	-	#N/A	#N/A	0	0	0	8720	2	1
**13862**	NSP12/RdRP	orf1ab	-	-	QHD43415.1:p.4533T>I; QHD43415.1:p.4533-	MODERATE; MODIFIER	0	13	0	8707	2	1
**14220**	NSP12/RdRP	orf1ab	-	-	QHD43415.1:p.4652D	LOW	0	1	0	8720	1	1
**15290**	NSP12/RdRP	#N/A	-	-	#N/A	#N/A	0	0	8721	0	1	1
**15344**	NSP12/RdRP	#N/A	-	-	#N/A	#N/A	0	0	0	8721	1	1
**15435**	NSP12/RdRP	orf1ab	-	-	QHD43415.1:p.5057-	MODIFIER	8720	0	0	0	2	1
**15669**	NSP12/RdRP	#N/A	-	-	#N/A	#N/A	0	8720	0	1	1	1
**16221**	NSP12/RdRP	orf1ab	-	-	QHD43415.1:p.5319P	LOW	0	0	8721	0	1	1
**16377**	NSP13/Helicase	orf1ab	-	-	QHD43415.1:p.5371P	LOW	0	5	8716	0	1	1
**17122**	NSP13/Helicase	orf1ab	-	-	QHD43415.1:p.5620A>T; QHD43415.1:p.5620A>S	MODERATE	1	0	8719	0	2	1
**17122**	NSP13/Helicase	orf1ab	-	-	QHD43415.1:p.5620A>T; QHD43415.1:p.5620A>S	MODERATE	1	0	8719	0	2	1
**17403**	NSP13/Helicase	orf1ab	-	-	QHD43415.1:p.5713A	LOW	0	2	0	8718	2	1
**17415**	NSP13/Helicase	orf1ab	-	-	QHD43415.1:p.5717A	LOW	0	8721	0	0	1	1
**17415**	NSP13/Helicase	orf1ab	-	-	QHD43415.1:p.5717A	LOW	0	8721	0	0	1	1
**17656**	NSP13/Helicase	orf1ab	-	-	QHD43415.1:p.5798M>V	MODERATE	8700	0	3	0	19	1
**17747**	NSP13/Helicase	orf1ab	B1a1	-	QHD43415.1:p.5828P>L; QHD43415.1:p.5828-	MODERATE; MODIFIER	0	856	0	7842	24	1
**17858**	NSP13/Helicase	orf1ab	B1a	-	QHD43415.1:p.5865Y>C	MODERATE	7845	0	876	0	1	1
**18060**	NSP14/Exonuclease	orf1ab	B1	-	QHD43415.1:p.5932L; QHD43415.1:p.5932-	LOW; MODIFIER	0	886	0	7832	4	1
**18078**	NSP14/Exonuclease	orf1ab	-	-	QHD43415.1:p.5938K	LOW	1	0	8720	0	1	1
**18312**	NSP14/Exonuclease	orf1ab	-	-	QHD43415.1:p.6016V	LOW	1	2	0	8718	1	1
**18312**	NSP14/Exonuclease	orf1ab	-	-	QHD43415.1:p.6016V	LOW	1	2	0	8718	1	1
**18573**	NSP14/Exonuclease	orf1ab	-	-	QHD43415.1:p.6103S	LOW	0	8720	0	1	1	1
**19644**	EndoRNAse	#N/A	-	-	#N/A	#N/A	0	8697	0	0	25	1
**19861**	EndoRNAse	orf1ab	-	-	QHD43415.1:p.6533A>T	MODERATE	1	0	8681	0	40	1
**19862**	EndoRNAse	orf1ab	-	-	QHD43415.1:p.6533A>V; QHD43415.1:p.6533-	MODERATE; MODIFIER	0	5	0	8676	41	1
**19875**	EndoRNAse	#N/A	-	-	#N/A	#N/A	0	0	0	8682	40	1
**20255**	EndoRNAse	orf1ab	-	-	QHD43415.1:p.6664-; QHD43415.1:p.6664D>G	MODIFIER; MODERATE	8720	0	0	0	2	1
**20429**	EndoRNAse	orf1ab	-	-	QHD43415.1:p.6722P>L	MODERATE	0	1	0	8720	1	1
**20580**	EndoRNAse	orf1ab	-	-	QHD43415.1:p.6772V; QHD43415.1:p.6772-	LOW; MODIFIER	1	3	8715	0	3	1
**20749**	O-ribose methyltransferase	#N/A	-	-	#N/A	#N/A	0	0	8721	0	1	1
**21707**	S-Protein	S	-	-	QHD43416.1:p.49H>Y	MODERATE	0	29	0	8687	6	1
**21989**	S-Protein	S	-	-	QHD43416.1:p.143-144VY>D; QHD43416.1:p.143V>F	MODERATE	0	1	8714	0	7	1
**21990**	S-Protein	S	-	-	QHD43416.1:p.143-144VY>V	MODERATE	0	8712	0	0	10	1
**22430**	S-Protein	#N/A	-	-	#N/A	#N/A	0	0	8518	0	204	1
**22458**	S-Protein	S	-	-	QHD43416.1:p.299T>I	MODERATE	0	1	0	8469	252	1
**23042**	S-Protein	S	-	-	QHD43416.1:p.494-	MODIFIER	0	8686	0	1	35	1
**23108**	S-Protein	#N/A	-	-	#N/A	#N/A	0	0	8678	1	43	1
**23660**	S-Protein	#N/A	-	-	#N/A	#N/A	0	0	8721	0	1	1
**23677**	S-Protein	S	-	-	QHD43416.1:p.705V	LOW	0	8721	0	0	1	1
**24166**	S-Protein	#N/A	-	-	#N/A	#N/A	8719	0	0	0	3	1
**24622**	S-Protein	S	-	-	QHD43416.1:p.1020-	MODIFIER	0	8719	0	0	3	1
**24694**	S-Protein	S	B1a1a	-	QHD43416.1:p.1044G	LOW	8634	87	0	0	1	1
**24904**	S-Protein	S	-	-	QHD43416.1:p.1114-; QHD43416.1:p.1114I	MODIFIER; LOW	0	5	0	8715	2	1
**25318**	S-Protein	S	-	-	QHD43416.1:p.1252S	LOW	0	0	0	8719	3	1
**25318**	S-Protein	S	-	-	QHD43416.1:p.1252S	LOW	0	0	0	8719	3	1
**25350**	S-Protein	S	A2a10	-	QHD43416.1:p.1263P>L; QHD43416.1:p.1263-	MODERATE; MODIFIER	0	51	0	8661	10	1
**25642**	ORF3a	ORF3a	-	-	QHD43417.1:p.84L	LOW	0	1	0	8720	1	1
**25793**	ORF3a	ORF3a	-	-	QHD43417.1:p.134R>H; QHD43417.1:p.134R>L	MODERATE	0	3	8706	0	13	1
**25826**	ORF3a	#N/A	-	-	#N/A	#N/A	8706	1	0	0	15	1
**25919**	ORF3a	ORF3a	-	-	QHD43417.1:p.176T>I	MODERATE	0	1	0	8711	10	1
**25979**	ORF3a	ORF3a	-	-	QHD43417.1:p.196G>V; QHD43417.1:p.196-	MODERATE; MODIFIER	0	149	8570	0	3	1
**27191**	M-Protein	M	-	Ter223Ter	QHD43419.1:p.223-; QHD43419.1:p.223*	MODIFIER; LOW	8717	0	2	0	3	1
**27195**	orf	#N/A	-	-	#N/A	#N/A	8720	0	0	0	2	1
**27208**	ORF6	ORF6	-	-	QHD43420.1:p.3H>Y; QHD43420.1:p.3-	MODERATE; MODIFIER	0	3	0	8716	3	1
**27364**	ORF6	ORF6	-	-	QHD43420.1:p.55E>*; QHD43420.1:p.55E>Q	HIGH; MODERATE	0	1	8720	0	1	1
**27384**	ORF6	ORF6	-	-	QHD43420.1:p.61D; QHD43420.1:p.61-	LOW; MODIFIER	0	8682	0	38	2	1
**27874**	ORF7b	Intergenic	-	-	QHD43422.1	MODIFIER,DISTANCE=20	0	0	0	8705	17	1
**28115**	ORF8	ORF8	-	-	QHD43422.1:p.74I	LOW	0	1	0	8703	18	1
**28115**	ORF8	ORF8	-	-	QHD43422.1:p.74I	LOW	0	1	0	8703	18	1
**28253**	ORF8	ORF8	-	-	QHD43422.1:p.120F>L; QHD43422.1:p.120F; QHD43422.1:p.120-; QHD43422.1:p.121I>X	MODERATE; LOW; MODIFIER; HIGH	3	5	2	8703	9	1
**28657**	N-capsid	N	-	-	QHD43423.2:p.128D; QHD43423.2:p.128-	LOW; MODIFIER	0	151	0	8569	2	1
**28863**	N-capsid	N	-	-	QHD43423.2:p.197S>L; QHD43423.2:p.197-	MODERATE; MODIFIER	0	149	0	8570	3	1
**29574**	ORF10	#N/A	-	-	#N/A	#N/A	0	8711	0	0	11	1
**29614**	ORF10	ORF10	-	-	QHI42199.1:p.19C	LOW	0	8	0	8703	11	1
**29614**	ORF10	ORF10	-	-	QHI42199.1:p.19C	LOW	0	8	0	8703	11	1
**29807**	3'UTR	#N/A	-	-	#N/A	#N/A	1	80	0	1	68	1

### Sample collection and molecular investigations

The nasopharyngeal and oropharyngeal swabs (in viral transport medium) were received at NCDC, Delhi through the Integrated Disease Surveillance Programme were subjected to viral inactivation followed by RNA extraction using QIAamp Viral RNA Mini Kit (Cat. No. 52906, Qiagen). Total RNA content in the elute was quantified using NanoDrop (Thermo Fisher Scientific). The 260/280 ratio ranged between 1.6–2.2 for the majority of the samples. To ensure that sub-optimal RNA samples are also included in the study, we made use of SuperScript IV (Cat. No. 18091050, Thermo Fisher Scientific, Waltham, MA, USA), for superior first strand cDNA synthesis and included them for sequencing.

### Molecular diagnosis of COVID-19

A quantitative reverse transcription (RT)-PCR assay was used on purified RNA for detection of SARS-CoV-2 in the samples. Quantitative RT-PCR was carried out using TaqMan assay chemistry on ABI7500 platform. The primer/probe concentrations and reaction conditions for diagnostics were as per the WHO protocols ( Corman *et al.*, 2020). Two target genes were used for diagnosis of SARS-CoV-2, envelope (E) gene for screening and RNA dependent RNA polymerase (RdRp gene) for confirmation. The positive samples were analyzed based on the country of origin (traveller), contact with positive case, geographical location (community), gender and age. Samples from each group were selected and further processed for WGS of the SARS-CoV-2.

### Whole genome sequencing of SARS-CoV-2

**cDNA synthesis:** Total RNA from SARS-CoV-2 positive samples were quantified using Nanodrop and 50 ng of the RNA was taken for double-stranded cDNA synthesis. Briefly, first strand cDNA was made using 1.0 μl of random hexamer (50 ng/μl), 1.0 μl of dNTPs (10 nM) and 13.0 μl of total RNA with volume adjusted with nuclease-free water (NFW), followed by incubation at 65°C for 5 mins and cooling on ice. To this, 4.0 μl of 5X SSRT IV Buffer, 1.0 μl of 100 mM DTT, 1.0 μl of ribonuclease inhibitor and 1.0 μl of SSRT IV enzyme (200 U/μl) was added (Cat. No. 18091050, Thermo Fisher Scientific, Waltham, MA, USA) with incubation at 23°C for 10 minutes, 50°C for 10 minutes and 80°C for 10 minutes. 1.0 μl of RNase H was added to this and incubated at 37°C for 20 mins. Next, 20.0 μl of first strand cDNA was heated at 95°C for 3 minutes after addition of 10 pmol of random primers, 10 μM dNTPs and 1X Klenow Buffer, followed by immediate cooling on ice. Soon after, 1.0 μl of Klenow Fragment (Cat. No. M0210S, New England Biolabs) was added with incubation at 37°C for 60 mins, 75°C for 10 mins and 4°C for 10 mins. This was followed by Ampure beads purification (Cat. No. A63881, Beckman Coulter) and quantification using Qubit dsDNA HS assay kit (Cat. No. Q32854, Invitrogen).

**Nanopore library preparation and sequencing:** A total of 100 ng of double stranded cDNA was taken for next generation sequencing (NGS) using a highly multiplexed PCR amplicon approach for sequencing on the Oxford Nanopore Technologies (ONT) (Oxford, United Kingdom) MinION using V3 primer pools (ARTIC Protocol, see https://artic.network/resources/ncov/ncov-amplicon-v3.pdf). Amplification was done using Takara LA Taq® DNA Polymerase Hot-Start Version (Cat. No. RR042B, Takara) along with 2.5 mM dNTPs, 10X Buffer II LA Takara, 10 μM primer pool, with the final volume adjusted to 25 μl using NFW. Primers were made into two pools, pool A and pool B, with 5 μl of each primer from the 100 μM primer stock. The stock (100 μM) was diluted with NFW in order to obtain a working stock of each pool at 10 μM. PCR was performed with initial denaturation at 98°C for 30 secs followed by denaturation at 98°C for 15 secs, 65°C for 5 mins, for a total of 35 cycles, with hold at 4°C. Post PCR pooling (pool A and pool B) of PCR amplicons was followed by purification using Ampure beads. Following purification, 1.0 μl of the library was run on a DNA1000 Agilent bioanalyzer (Cat. No. 5067-1504, Agilent) to check for a size of ~400 bp. Next, 125 ng of each sample was taken forward for End prep with NEBNext Ultra II End Repair/dA tailing module (Cat. No. E7546, New England Biolabs). The reaction mix was incubated on a thermal cycler at 20°C for 5 mins followed by 65°C for 5 mins. Following this, 1.5 μl of End prep DNA was taken forward for native barcode ligation using native barcodes (EXP-NBD104 and EXP-NBD114, ONT) and Blunt/TA Ligase master mix (Cat. No. M0367, New England Biolabs). The mix was incubated at room temperature for 15 mins followed by purification using Ampure beads. The purified product was used for adaptor ligation using Adapter Mix II and Quick T4 DNA ligase (Cat. No. M2200L, New England Biolabs). After adaptor ligation, it was purified using a combination of short fragment buffer and Ampure beads resulting in a sequencing ready library. Library quantification was conducted using the Qubit dsDNA HS assay kit (Cat. No. Q32854, Invitrogen) and 70 ng of the library was used for sequencing. Barcoding, adaptor ligation, and sequencing were performed on samples with Ct values between 16–31. A ‘no template control’ was created at the cDNA synthesis step and amplicon generation step to detect cross-contamination between samples. Controls were barcoded and sequenced with both the high- and low-titer sample groups. The sequencing flowcell was primed and used for sequencing using MinION Mk1B.

### Illumina library preparation and sequencing

A common pool of cDNA was used for making both Illumina and Nanopore sequencing libraries and subsequent sequencing. cDNA (100 ng) was used to construct the Illumina library using theNextera XT protocol, as per manufacturer’s instructions (15031942 v05, Illumina Inc). Briefly, tagmentation of cDNA was done which tagged and fragmented the cDNA by addition of amplicon tagment mix (ATM) and tagment DNA buffer, as per manufacturer’s protocol, Illumina Inc with incubation at 55°C for 5 mins with heated lid option. Tagmentation was stopped by addition of neutralization tagment buffer. This was followed by the addition of unique index adapters (i7 and i5 adapters) to the samples. Index adapters are then used for PCR amplification at 72°C for 3 mins, 95°C for 30 secs and 12-cycles of 95°C for 10 secs, 55°C for 30 secs, 72°C for 30 secs; and 72°C for 5 mins. The PCR product was purified using AgencourtAMPure XP beads. The quantity of the sequencing ready library was measured using Qubit dsDNA HS assay kit (Cat. No. Q32854, Invitrogen) and quality by Agilent DNA HS kit (Cat. No. 5067-4626, Agilent). Illumina’s MiSeq platform was used for sequencing.

### Analysis pipeline for nanopore sequencing data

The raw fast5 files were base-called and demultiplexed using the Guppy basecaller (version 3.5.2). The fastq files were normalized by read length, thereby eliminating possible chimeric reads. Pre-alignment quality control was carried out to assess the read quality using Nanopack tools (version 1.29.0) ( De Coster *et al.*, 2018). Minimap2 (version 2.17) has been used to align the raw reads with the reference ( MN908947.3) ( Li, 2018). Nanopolish were used for accurate variant calling from the aligned output ( Loman, 2015) with options, minimum flanking sequence - 10, ploidy - 1 and minimum candidate frequency - 0.15. The possible heterozygous variants are filtered out as a separate group after the variants have been called. Post-alignment QC was then performed with Nanopack tools as well as the seaborn (version 0.10.1) package in python to create the distribution of amplicon quality and CT-value vs coverage and depth. Finally, a consensus fasta was created, wherein genomic regions with low coverage and low quality were masked using BCFtools (version 1.9).

### Miseq data analysis

The raw reads from the miseq were quality-checked by FASTQC (version 0.11.8). Trimgalore was used to trim the reads containing bad quality and the minimum length of 40 base pairs was kept as a threshold for the reads. HISAT2 is used to map the reads to the human genome (GRCh37) to remove potential the human rRNA reads for the contamination with default parameters [ Kim *et al.*, 2019]. The unmapped reads from the human are converted from bam to fastq using bam2fastq (version 2.29) to align to the SARS-CoV-2. HISAT2 was used to align unmapped reads with the SARS-CoV-2 reference genome (MN908947.3 build). Using both samtools (version 1.9) and BCFtools from the SARS-CoV-2 aligned bam files the consensus fasta was generated. The variants in the samples were called using BCFtools and VarScan.

### Phylogeny and network analysis

The fasta sequences were aligned using MAFFT (version 7.455) considering the MN90894.3 version as the reference sequence. Phylogenetic trees were constructed using the Neighbour joining algorithm as statistical method and maximum composite likelihood as model in MEGA X software. FIGTREE (version 1.4.4) was used for the graphical visualisation of phylogenetic analysis. Pheatmap (version 1.0.12) and ComplexHeatmap (version 1.10.2) packages from R 3.6.2 were used to plot the heatmaps. Haplotype network analysis was conducted using PopART (version 1.7) [ Leigh & Bryant, 2015].

### Protein based annotation

In order to categorize the specific amino acid change and the proteins containing the variants, they were annotated with SnpEff (version 4.5) [ Cingolani *et al.*, 2012]. The annotation was performed according to the known reference genome of SARS-CoV-2 (i.e. NC_045512) in the NCBI database [ Wang *et al.*, 2020]. SARS-CoV-2 polypeptide ORF1ab encodes 16 non-structural proteins (nsp) as a result of proteolytic processing. Hence, for better mapping of the variants present in ORF1ab, we annotated the variants according to the respective nsp residue number.

Further, conservation analysis of the full-length sequences of proteins harbouring these mutations was done on the basis of the six other coronaviruses. The multiple sequence alignment of seven protein sequences was performed with Clustal Omega [ Madeira *et al.*, 2019]. The conservation score of ORF3a and ORF8 were calculated with low confidence due to introduced gaps at these positions during alignment. The amino acid type was defined as hydrophobic (G, A, V, L, I, M, P, F, W), polar (S, T, N, Q) or charged (H, K, R, D, E). With this definition, the type of change of residues was calculated.

### Three-dimensional protein models

To map the high frequency mutations on proteins, we took protein structure models of SARS-CoV-2 from the Swiss Model repository ( https://swissmodel.expasy.org/repository/species/2697049) [ Waterhouse *et al.*, 2018] and models were generated through comparative modeling and by using Robetta prediction server [ Kim *et al.*, 2004]. In total three structural models were obtained from swiss model repository and the nsp12 structure was obtained from RCSB [PDB ID 6M71] [ Gao *et al.*, 2020]. The details of missing residues or structural domains of each protein is described below. The Spike protein exists as a homotrimer consisting of 1273 residues in each chain with a total of 3819 amino acids. While electron microscopy structures are available for different conformations of Spike protein, in particular S1 region, many residues within the S1 and S2 stalks are missing [ Walls *et al.*, 2020; Wrapp *et al.*, 2020]. Therefore, to map the mutations onto the structure we obtained the S1 stalk model of the spike protein (residues 15-1137). Similarly, nsp3 also known as PL-PRO (papain-like proteinase) is a large multi-domain transmembrane protein. For mapping the nsp3 mutations, we considered the model for the nucleic acid binding domain (residue 1089-1203), which is conserved in betacoronaviruses [ Angelini *et al.*, 2013]. The Nucleocapsid protein comprise of N-terminal and C-terminal domains connected by linker region. However, structural information for the linker region is unknown.

## Results

### Demographic details and travel history of the subjects

The majority of the SAS-CoV-2-positive samples were obtained from New Delhi, covering the national capital region of Delhi, India and a few clusters identified by the surveillance team (covering the states of Delhi, Tamil Nadu, Maharashtra, Uttar Pradesh, Andhra Pradesh, West Bengal, Bihar, Orissa, Rajasthan, Haryana, Punjab, Assam and Union territory of Ladakh). The mean (standard deviation) age of the total 127 subjects was 41.4±17.5 years with age range 0.5–76 years and median of 39 years. The male-to-female gender ratio of in the age group <39 years was 35:28, while the remaining 46 subjects >40 years had the ratio of 58:6. Exposure to COVID-19 was suggestive of travel history of subjects to Europe, West Asia and East Asia. A minority of subjects were from foreign countries: Indonesia (n=14), Thailand (n=2) and Kyrgyzstan (n=2). The identified localities of the subjects will further help in molecular surveillance of SARS-CoV-2 in respective geographical regions.

### Profile of SARS-CoV-2 genome sequences

The average amplicon coverage for the V3 ARTIC primers used in the study was more than 1000X coverage across the majority of the samples ( *Extended data*, Supplementary figure S2 [ Kumar *et al.*, 2020b]). We also looked into whether lower Ct values are a good indicator of genome coverage using a minimal set of virus mapping reads. We plotted genome coverage and average sequencing depth across Ct value of both the genes (E and RdRp). It was observed that higher Ct values (27 onwards) have increased possibility of lower genome coverage ( *Extended data*, Supplementary figure S2 [ Kumar *et al.*, 2020b]), although some lower Ct value samples also had incomplete genome coverage. We sequenced a subset of samples on orthogonal platforms and sequencing methods (shotgun and amplicon) using ONT and Illumina platform. Significantly, we observed that the genetic variants were common between both the platforms.

### NGS analysis and construction of phylogeny network for SARS-CoV-2 sequences

**Figure 1.**
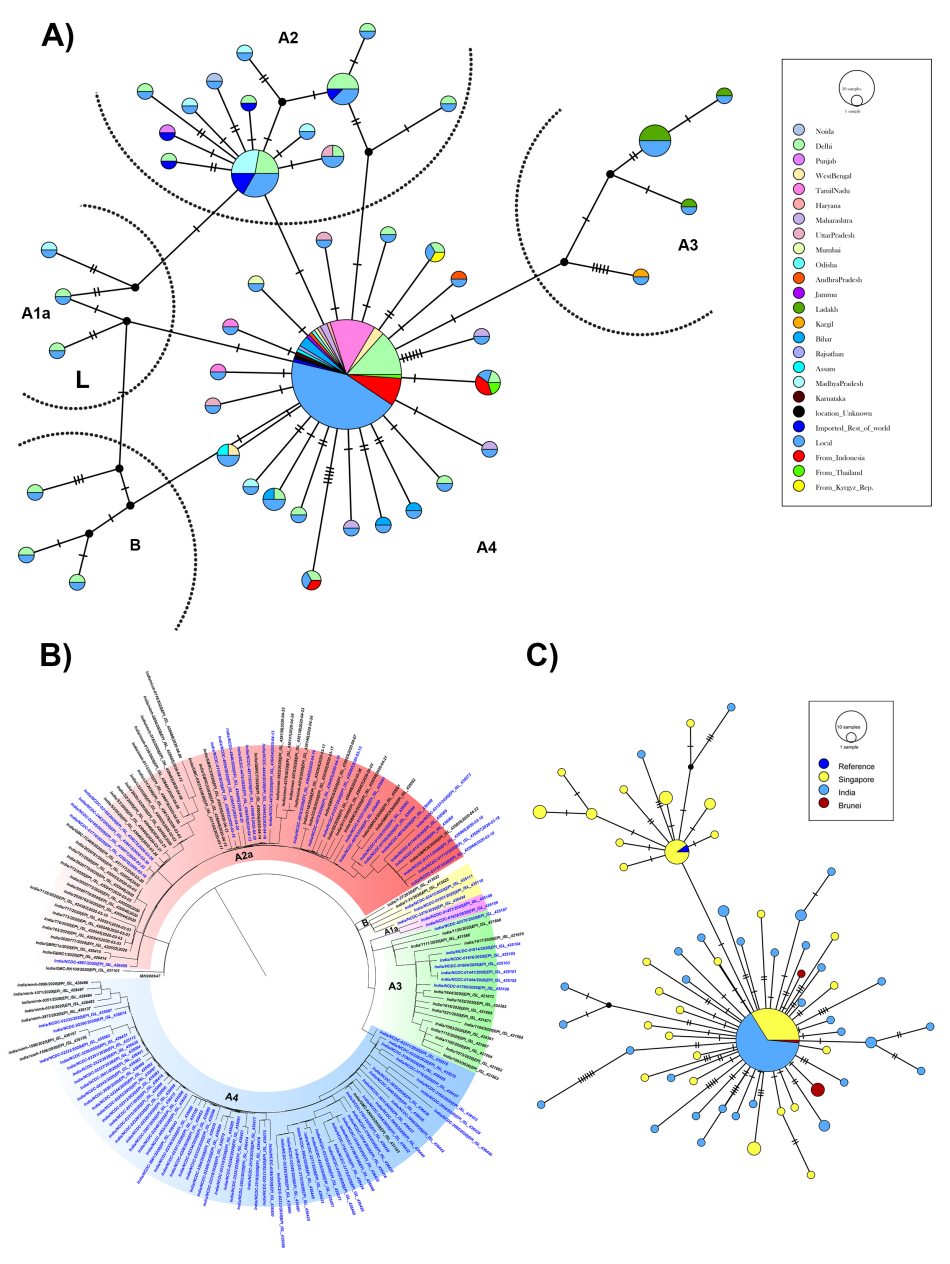
Haplotype network and phylogenetic analysis of SARS-CoV-2 sequences. ( **A**) The network analysis (integer neighbourhood joining network at reticulation tolerance value of 0.5, popART) of SARS-CoV-2 sequences from this study showing distinct clades with their geographical locations. A4 clade described in this study for the first time has widespread geographical affiliations. A3 being more confined to Ladakh and mostly these clusters represent introduction of the infection through travel history. Mutations are marked with hatch lines connecting the nodes. ( **B**) Phylogenetic tree (generated using MEGA) of SARS-CoV-2 genomes from sequences submitted from across India depicting major clade-based distribution of SARS-CoV-2 in India. ( **C**) Network analysis (Median spanning network) of A4 clade sequences submitted from Indian and neighbouring East Asians countries.

A total of 104 samples passed the quality threshold for mapping full genome coverage threshold for SARS-CoV-2 genome <0.05 N content with median coverage ~1500× (see *Underlying data* for each accession number [ Kumar *et al.*, 2020a]. A total of 23 samples that did not qualify the threshold criteria were excluded from strain identification. The phylogenetic analysis of 104 high quality sequences reveal all the strains to be grouped into two major clades, a sub-clade and other clades ( Figure 1 and *Extended data*, Supplementary figure S3 [ Kumar *et al.*, 2020b]). From variants perspectives, we observed 163 variants representing singletons (107 variants), rare: 2-5% (45 variants), and common variants: >5% (11 variants). The common variants observed were 241 (Leader sequence), 3037 (NSP3), 6310 (NSP3), 6312 (NSP3), 11083 (NSP6), 13730 (NSP12/RdRp), 14408 (NSP12/RdRp), 23403 (S-Protein), 23929 (S-Protein), 25563 (ORF3a), 28311 (N-capsid). The following cluster-based segregation of SARS-CoV2 sequences was observed.

**Cluster 1:** The sequences (n=26) in this cluster belonged to the G-clade [variant 23403 (Spike protein D614G) as per GISAID nomeclature (Global Inititative on Sharing All Influenza Data)] Based on Nextstrain classification these 26 sequence belonged to A2a clade [(denoted by positions C241T; C3037T, A23403G (S: D614G); C14408T (ORF1b/RdRp: P314L)] ( Figure 1A). The additional frequent variants in this cluster observed were 25563 (ORF3a, n=9),18877 (NSP14/Exonuclease, n=7), 26735 (M-protein, n=6), 22444 (S-protein, n=5), and 28854 (N-capsid, n=5). One novel variant 1947 T>C (NSP2) was observed in two strains in this cluster.

**Cluster 2:** In our study large numbers of strains (n=65) belong to this unclassified cluster (as per GISAID and Nextstrain). The strains in this cluster had the predominant variants, G11083T variant (NSP6) (n=65), C13730T in RdRp (n=65), C28311T (N-capsid); n=65, C6312A (NSP3 variant); n=64 (one sequence being called N), C23929T (S-protein), n=50 (other being low depth/N bases). The variant C6310A (NSP3); n=22 being observed as another frequent alteration ( Figure 1B). We also observed few novel variants, G12685T ( *NSP8*); n=5 and TC1706T ( *NSP2)*; n=4, T7621C ( *ORF1a/b*); n=3, A21792T (S protein); n=3, G13920A *(NSP12/RdRp)*; n=2, *A16355G (NSP13*/Helicase); n=2 and G18803T ( *NSP14*/Exonuclease); n=2 in this cluster. The majority of the key cluster variants 11083, 13730, 28311, 6312, 23929 are also shared in sequences submitted from Singapore and Brunei; additionally, similar clade sequences were observed in India submitted by National Institute of Mental Health and Neuro-Sciences (NIMHANS) and Gujarat Biotechnology Research Centre (GBRC) cohort (see *Underlying data* for details of all accession numbers [ Kumar *et al.*, 2020a]. Based on the geographical location of the subjects of this cluster, a considerable number of Indonesians (n=7) and two each from Thailand and Kyrgyzstan were part of this cluster from our study site. This probably suggests introduction of this particularly from East Asian countries into India.

**Cluster 3:** This subclass of strains (n=7) harbouring a common variant G11083T (NSP6), G1397A (NSP2) and T28668C (N-capsid) are described for the A3 clade (Nextstrain) in additions to G29742T ( Figure 1A). Other mutated positions, i.e. C884T (NSP2), G8653T (NSP4) were observed in 5 samples, whereas T16993C (NSP13), n=4; T25461C (ORF3a), n=4 and A27191G (M-protein), n=2 are putative novel sites.

The phylogeny analysis of these clusters segregated with the other Indian SARS-CoV-2 genome sequences as recently reported (GISAID) ( Figure 1B).

**Other SARS-CoV-2 genomes**: Two SARS-CoV-2 belonging to the A1a clade had a SNP profile of 11083(NSP6)/14805 (NsP12/RdRp)/2480 (NSP2)/2558 (NSP2)/26144 (ORF3a). In addition, we observed three B clade sequences having position 8782 C>T (NSP4) and 28144 T>C (ORF8; S clade GISAID) mutated and with one sequence with an additional C18060T B1 variant. One genome from Maharashtra had no variants and probably represented the first genome sequenced from Wuhan, China.

### Redefining cluster 2 with neighbourhood re-joining

**Figure 2.**
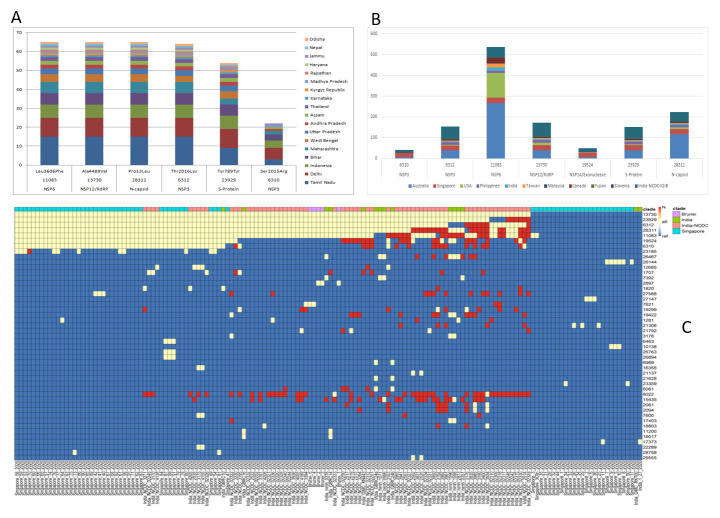
Distribution of A4 clade within India and globally. ( **A**) Distribution of A4 clade variants across different geographical regions from the cohort. ( **B**) Distribution of A4 clade variants across different geographical regions across the globe. ( **C**) Comparison of A4 cluster sequences across the South-East Asian region showing sharing of variants and haplotype across South-East Asian region.

With over represented variants in cluster 2 for variants 11083/13730/28311/6312/23929, we defined this cluster with A4 clade. This has similarity with sequences submitted from Singapore, Brunei and other Indian sequences submitted. The haplotype network analysis suggests that these sequences are having a common origin from East Asia/South-East Asia ( Figure 1C and Figure 2). This A4 clade has multiple variants in important region of viral genome, RdRp (A97V), N-capsid (P12L), NSP3 (T2016K), NSP6 (L37F) and NSP3 (S1197R) variants. In our cohort of samples, the majority of subjects were from Tamil Nadu, Delhi and Indonesia and others were from various other states ( Figure 2).

### Protein-wise analysis of SARS-CoV-2 variants

To provide quantitative insights into the mutant proteins, we characterized amino acid substitutions across the 104 viral genomes. Of the 53 point mutations identified, 29 were missense that resulted in amino acid substitutions. *Extended data*, Supplementary figure S4 [ Kumar *et al.*, 2020b] plots the occurrence of these mutations as a function of each viral protein. The frequency of amino acid variations was highest in nsp6 (L37F), present in 68 genomes, followed by nsp12 (A97V) in 65, nsp3 (T1198K) in 62 and nucleocapsid (P13L) in 53 genomes. Interestingly, the D614G mutation in spike protein, which is considered as a prevalent global mutation [ Bhattacharyya *et al.*, 2020; Korber *et al.*, 2020], was present in only 26 of the 104 sequenced genomes.

The analysis of occurrence of each mutation with the type of amino acid change have shown that ~45% of these are synonymous changes ( *Extended data*, Supplementary figure S5 [ Kumar *et al.*, 2020b]). Within frequently occurring mutations, P13L, L37F, A97V also showed no major residue alterations. However, T1198K in nsp3 involve acquisition of a charged group along with the key S protein mutation (D614G) also involves loss of the charged group. These mutations that lead to positively charged groups may cause more severe structural and functional effects.

We also compared SARS-CoV-2 mutation sites with other six coronavirus sequences ( *Extended data*, Supplementary figure S5b [ Kumar *et al.*, 2020b]). Most of the mutations were present in variable locations. Out of 29 mutations, 10 are present on highly conserved residue locations. Interestingly, a higher frequency of mutations are at positions that evolve faster/are variable across the coronaviruses, except for A97L and L37F, which are present on conserved locations.

**Figure 3.**
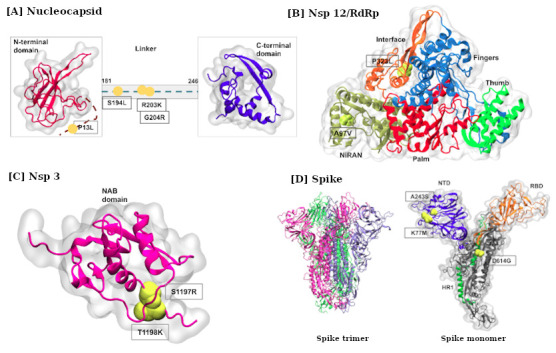
Mapping of high frequency mutations on proteins displaying top-most mutations in SARS-CoV2. ( **A**) Nucleocapsid, the N- and C-terminal domains are coloured in red and purple, respectively, where the missing linker region is shown as dotted line. ( **B**) NSP12/RdRp, showing the five of its domains, NIRAN (olive), Palm (red), Thumb (green), Fingers(blue) and Interface(orange). ( **C**) Nucleic acid-binding domain (NAB) of Nsp3 protein shown in pink color. ( **D**) The trimeric spike protein showing 3 chain like structure. The right panel shows a single chain where the NTD, RBD and HR1 domains are coloured in orange, purple and green, respectively, whereas the rest of the structure is coloured grey. The mutations in all the proteins are marked within black boxes shown in yellow spheres.

The structural analysis of different viral proteins, nucleocapsid, nsp3, nsp12, and spike protein was conducted and analysis of nucleocapsid protein [ Kang *et al.* 2020] showed its variants were present in the linker region ( Figure 3A). The observed mutations in nsp12 (a highly conserved protein) are overlaying onto the interface (P323L) and NiRAN (A97L) region. The latter is critical as it contains a Zn+ binding site; however, little is known about the exact functional output. In contrast, the P323L mutation is present on protein interaction junctions where a hydrophobic cleft is known to bind to inhibitors ( Figure 3B).

The amino acid change from proline to leucine may result in significant backbone changes, due to the absence of unique proline-induced distortions in the protein backbone. Next, we mapped mutations within Nsp3 protein ( Figure 3C). In particular, the mutations were present on the NAB domain of nsp3, which is a nucleic acid binding domain and also interacts with nsp12 [ Jian *et al.*, 2018]. This mutation may impact RNA synthesis machinery; however, little is known about its exact mechanism of action. Lastly, the D614G mutation in spike protein is an interesting substitution and has been reported with increased tally ( Figure 3D) [ Bhattacharyya *et al.*, 2020; Korber *et al.*, 2020]. Structurally, this mutation is located in the S1 subunit that also contains the RBD domain. Although present outside the functional region, the proximity of D614G around S1 cleavage site implicates an important change in the local environment.

## Discussion

This is the first comprehensive genomic picture of the SARS-CoV-2 prevalent in the Indian population during the early phase of outbreaks. The understanding is important keeping in view the vast geographical expanse and population density of India. There were three major waves of viral entry in India associated with multiple outbreaks ( *Extended data*, Supplementary figure S6 [ Kumar *et al.*, 2020b]). The first wave includes importation of SARS-CoV-2 (A2a cluster) through travelers from Europe (Italy, UK, France, etc.) and the USA. Second wave of SARS-CoV-2 (A3 cluster) was linked with the Middle East (Iran and Iraq). The third wave comprises combined viral (haplotype redefined as A4) entries from Southeast Asia (Indonesia, Thailand and Malaysia) and Central Asia (Kyrgyzstan). The study, taken together with those of other reported genomes ( Potdar *et al.*, 2020), revealed that the A4 cluster (previously unclassified) is the most prevalent in the available genome sequences from India. The observed distinct A4 genome lineage of SARS-CoV2 in the Indian Subcontinent, which is present in East Asian Countries like Singapore and Indonesia, may allow further research and investigation to understand the evolution of SARS-CoV-2 genomes in Southeast Asian countries. Many novel mutations identified may be specific to Indian conditions, but more genomic data is needed to strengthen the assumption to rule out sampling bias and other factors ( Lu *et al.*, 2020). However, a more detailed analysis of these genomes might provide information whether these variations need to be considered during design of diagnostic primers as the need for testing shoots up. It may allow for creation of cost-effective panels to trace the movement of lineage specific strains across geographical regions more rapidly and effectively. Lots of efforts are ongoing to identify suitable vaccine candidates through docking studies. These observations are important to consider the variants that map to the Indian genomes during such prioritization studies, since these strains would now form a major fraction of the genomes that are likely to become more prevalent in India after lockdown. Mapping of these variant genomes in conjunction with the clinical history in terms of recovery, hospitalization and co-morbidity might allow identification of variants that should be actionable and would also have relevance for prognosis. It is imperative that robust genomic data based on large sample size, including rural populations with even distribution can bring out the real scenario once correlated with epidemiological data eventually helping in drafting of further management policies.

## Data availability

### Underlying data

All sequence data have been deposited with GISAID [ Shu & McCauley, 2017].

Figshare: Acknowledgement table GISAID and Accession. https://doi.org/10.6084/m9.figshare.12624815.v4 [ Kumar *et al.*, 2020a].

This project contains all GISAID accession numbers generated and analysed in this study.

### Extended data

Figshare: Supplementary figures S1-S6. https://doi.org/10.6084/m9.figshare.12631880.v1 [ Kumar *et al.*, 2020b].

This file contains the following extended data:

- Supplementary Figure S1: A schematic diagram showing numbers of samples with their geographical affiliations with respect to states of India.
- Supplementary Figure S2: Sequencing data quality parameters and orthogonal platform validation.
- Supplementary figure S3: A heat map representation of SARS-CoV-2 variants per sample and their respective segregation in respective clusters.
- Supplementary Figure S4: Protein annotation of the amino acid substitutions.
- Supplementary Figure S5: Amino acid properties marked as a function of mutations.
- Supplementary Figure S6: Scheme showing importation of prevalent SARS-CoV-2 genomes (3 major waves) in India.

Data are available under the terms of the Creative Commons Attribution 4.0 International license (CC-BY 4.0).

## Author information

Pramod Kumar, Rajesh Pandey, Pooja Sharma and Mahesh S. Dhar are joint first authors. Mohammed Faruq, Anurag Agrawal and Partha Rakshit are joint senior authors.
