# Supplementary material for: Integrated genomic view of SARS-CoV-2 in India
Source: Wellcome Open Res. 2020 Aug 3;5:184. [Version 1] doi: 10.12688/wellcomeopenres.16119.1 (PMC7506191; doi:10.12688/wellcomeopenres.16119.1)
Supplement: Peer review history

# Peer review: Reviewer response for version 1

**DOI:** 10.21956/wellcomeopenres.17692.r40421

**Published:** 2020-09-28

**Authors:** Sushil Kumar Sharma

**Affiliations:** 1 Division of Virology, Defence Research and Development Establishment, Gwalior, Madhya Pradesh, India

This is an extensive study revealing the comprehensive genomic picture of the SARS-CoV-2 prevalence in the Indian population during the early phase of outbreaks explaining the three major waves of viral entry in India associated with multiple outbreaks *i.e.* first wave (A2a cluster from Europe and USA), second wave (A3 cluster from Middle East); and the third wave (A4 cluster from Southeast Asia) of importation of SARS-CoV-2 from various countries. The study has been designed and executed meticulously with logical conclusions and publication-worthy merit.

  

1. **Results: Demographic details and travel history of the objects:** “The majority of the SAS-CoV-2…” may please be replaced with “ The majority of the SARS-CoV-2…”
2. The Supplementary Figure S6: “Scheme showing importation of prevalent SARS-CoV-2 genomes (3 major waves) in India” may be included in the main text.
3. D614G mutation has been reported in several studies. For example in one of the studies it has been claimed by Mr Babu V Bassa that the frequency of G614 variant in India is at least 90% as of June, 2020 (Bassa and Uppu, 2020 ^1^) as may be seen at the biorxiv.org site ( https://www.biorxiv.org/content/10.1101/2020.06.04.128751v1). This data from different regions of India needs to be discussed for better comprehension.

Is the work clearly and accurately presented and does it cite the current literature?

Yes

If applicable, is the statistical analysis and its interpretation appropriate?

I cannot comment. A qualified statistician is required.

Are all the source data underlying the results available to ensure full reproducibility?

Yes

Is the study design appropriate and is the work technically sound?

Yes

Are the conclusions drawn adequately supported by the results?

Yes

Are sufficient details of methods and analysis provided to allow replication by others?

Yes

Reviewer Expertise:

Molecukar biology,

I confirm that I have read this submission and believe that I have an appropriate level of expertise to confirm that it is of an acceptable scientific standard.

## References

[1] : The Frequency of G614 SARS-CoV-2 Variant in India.2020; 10.20944/preprints202008.0148.v1 10.20944/preprints202008.0148.v1

# Peer review: Reviewer response for version 1

**DOI:** 10.21956/wellcomeopenres.17692.r40420

**Published:** 2020-09-21

**Authors:** Suresh Singh Yadav

**Affiliations:** 1 Department of Biological Regulation, Weizmann Institute of Science, Rehovot, Israel

The study entitled ‘Integrated genomic view of SARS-CoV-2 in India’ explores the comprehensive genomic analysis of the SARS-CoV-2 early outbreaks in India. The study is highly relevant to the current epidemic and may add important information for further research and strain evolution of SARS-CoV-2, especially in India. Along with other clades previously known (named A2a, A2 and A3….), the authors reported that A4 (previously unclassified) is the most prevalent in the available genome sequences from India. The authors discussed that several novel mutations identified may be exclusive to the Indian population/conditions. It can also be helpful to trace the movement of lineage-specific strains across geographical regions more efficiently. The study is well designed, executed and concluded, and has significant merit to be published. The authors may address the following minor comments before indexing:

1. The authors should mention what was the positive and negative control for the qPCR.
2. Abstract - Methods: Sentence may be refreshed into two small sentences.
3. Methods: subheading ‘Sample collection and molecular investigations’ line seven - may be as ‘Absorbance ratio (260/280 nm) was ranged between 1.6–2.2 for most of the samples indicating its good quality

Is the work clearly and accurately presented and does it cite the current literature?

Yes

If applicable, is the statistical analysis and its interpretation appropriate?

Yes

Are all the source data underlying the results available to ensure full reproducibility?

Yes

Is the study design appropriate and is the work technically sound?

Yes

Are the conclusions drawn adequately supported by the results?

Yes

Are sufficient details of methods and analysis provided to allow replication by others?

Yes

Reviewer Expertise:

Molecular biology, cell signalling, epigenetics

I confirm that I have read this submission and believe that I have an appropriate level of expertise to confirm that it is of an acceptable scientific standard.

# Peer review: Reviewer response for version 1

**DOI:** 10.21956/wellcomeopenres.17692.r39810

**Published:** 2020-08-24

**Authors:** Vijay Nema

**Affiliations:** 1 Division of Molecular Biology, ICMR-National AIDS Research Institute, Pune, Maharashtra, India

This manuscript is well designed and executed to explore importation of SARS-CoV-2 genome with respect to major clade depicting A2a, A3 and A4 wave to India however, SARS-COV-2 Cluster B as well as A1a though minor is not mentioned for its entry in India and the Indian subcontinent. 

 The following amino acid substitutions are reported predominantly in Orf1ab region of the SARS-CoV-2 genome and its occurrence is in the following order L37F > A97V > T1198K > D614G > P323L > S1197R, however only D614G from spike region seems to be showing more infectivity and transmission efficiency in Europe while rest of the amino acid substitutions need to be explored for their infectivity and transmission efficiency. This will provide significant association between different mutation patterns and transmission profile with respect to India and the Indian subcontinent.

Is the work clearly and accurately presented and does it cite the current literature?

Yes

If applicable, is the statistical analysis and its interpretation appropriate?

I cannot comment. A qualified statistician is required.

Are all the source data underlying the results available to ensure full reproducibility?

Yes

Is the study design appropriate and is the work technically sound?

Yes

Are the conclusions drawn adequately supported by the results?

Yes

Are sufficient details of methods and analysis provided to allow replication by others?

Yes

Reviewer Expertise:

Molecular Biology, Metagenomics, Tuberculosis drug discovery

I confirm that I have read this submission and believe that I have an appropriate level of expertise to confirm that it is of an acceptable scientific standard.
